# The role of red ginseng in men’s reproductive health: a literature review

**DOI:** 10.1186/s12610-023-00203-0

**Published:** 2023-10-26

**Authors:** Hao Wang, Jiwei Zhang, Dongyue Ma, Ziwei Zhao, Bin Yan, Fu Wang

**Affiliations:** Department of Andrology, Xiyuan Hospital of China Academy of Chinese Medical Sciences, Beijing, 100091 China

**Keywords:** Red ginseng, Korea, Men’s reproductive health, Male infertility, Erectile dysfunction, Prostate diseases, Ginseng rouge, Corée, Santé reproductive des hommes, Infertilité masculine, Dysfonction érectile, Maladies de la Prostate

## Abstract

**Background:**

Red ginseng (RG) is a traditional herb commonly used in China, Korea, and other East Asian countries. Recently, it has demonstrated a better clinical value in men’s reproductive health (MRH). The present review aimed to examine the effects of RG treatment on MRH.

**Results:**

Overall, 42 articles related to RG application in MRH were reviewed, of which 31 were animal experiments and 11 were clinical studies. Furthermore, this review analyzed the use of RG in some male reproductive diseases in clinical trials and determined the associated mechanisms of action. The mechanism of action of RG in MRH may be related to oxidative stress, regulation of sex hormones and spermatogenesis-related proteins, and anti-inflammation.

**Conclusions:**

The application of RG for the treatment of male infertility, erectile dysfunction, and prostate diseases has the potential to contribute to MRH.

**Supplementary Information:**

The online version contains supplementary material available at 10.1186/s12610-023-00203-0.

## Résumé

**Contexte:**

Le ginseng rouge est une herbe traditionnelle couramment utilisée en Chine, en Corée et dans d'autres pays d'Asie de l'Est. Récemment, il a démontré une valeur clinique en santé reproductive des hommes. La présente revue visait à examiner les effets du traitement par ginseng rouge en santé reproductive masculine.

**Résultats:**

Au total, 42 articles liés à l’utilisation du traitement par ginseng rouge en situation d’altérations de la santé reproductive mâle ont été examinés, dont 31 étaient des expériences sur des animaux et 11 des études cliniques. De plus, cette revue a analysé l'utilisation du ginseng rouge dans certaines maladies de la reproduction masculine lors d’essais cliniques, et a déterminé les mécanismes d'action associés. Le mécanisme d'action du ginseng rouge en santé reproductive mâle peut être lié au stress oxydatif, à la régulation des hormones sexuelles et des protéines liées à la spermatogenèse, et à une action anti-inflammatoire.

**Conclusions:**

L'application du ginseng rouge pour le traitement de l'infertilité masculine, de la dysfonction érectile et des maladies de la prostate a le potentiel de contribuer à la santé reproductive des hommes.

## Background

With a recent increase in the prevalence of male diseases, men’s reproductive health (MRH) has received an increasing attention [1]. A broad definition of MRH includes both pathophysiological and psychosocial issues [2]. MRH is related to the erectile function, testicular functions of steroidogenesis (testosterone synthesis), and spermatogenesis, and it is influenced by numerous factors [3, 4]. These factors include unhealthy eating habits, delayed marriage, exposure to environmental pollutants, psychological stress, drug abuse, and natural aging in humans [5–8]. Given the influence of traditional sexual concepts, many men are reluctant to admit that they have a reproductive disorder. Furthermore, some patients are concerned about the adverse effects of oral therapeutic drugs or have unrealistic perceptions regarding current treatments, hindering them from seeking healthcare [9]. Given this background, a series of food products focusing on MRH have emerged and are gradually being accepted by patients [10]. However, these functional food products differ from therapeutic drugs, and their exact therapeutic effects on male reproduction-related diseases remain uncertain.

Red ginseng (RG) is a cooked product of ginseng that undergoes several processes, such as infiltration, cleaning, sorting, steaming, and drying, and it is widely used as an herbal medicine in some East Asian countries. Many clinical studies and animal experiments have reported that Korean RG has beneficial effects on MRH, including the regulation of testicular, erectile, and prostate functions [11–13]. However, currently, there is a lack of systematic review of these studies, although a few reviews tend to report the efficacy and safety of Korean RG in male diseases by meta-analysis [14]. Moreover, to date, these studies have not been analyzed in detail from a holistic perspective. In the present study, we systematically reviewed the existing relevant literature, analyzed the efficacy of RG in MRH through clinical evidence, and elaborated its related mechanisms along with basic research to provide a reference for the future application of RG in MRH.

## Materials and methods

### Search strategy

The PubMed and Web of Science databases were searched for relevant studies (up to December 2022). We also reviewed the reference lists of the studies identified via our search strategy and selected those that seemed relevant according to our keywords. We set out relevant search terms by referring to previous systematic reviews on MRH [15, 16]. The following search terms were used in Table 1.

**Table 1:** PubMed search strategy

No. Search item
#1 Korean red ginseng [Title/Abstract]
#2 red ginseng [Title/Abstract]
#3 red ginseng extract [Title/Abstract]
#4 Or/#1-#3
#5 reproductive [Title/Abstract]
#6 reproductive health [Title/Abstract]
#7 reproduction [Title/Abstract]
#8 male infertility [Title/Abstract]
#9 fertility [Title/Abstract]
#10 erectile dysfunction [Title/Abstract]
#11 sexual dysfunction [Title/Abstract]
#12 testis [Title/Abstract]
#13 prostate [Title/Abstract]
#14 penis [Title/Abstract]
#15 Or/#5 - #14
#16 #4 and #15

### Eligibility criteria

#### Inclusion criteria

We comprehensively and systematically searched for studies in accordance with the criteria outlined in the Preferred Reporting Items for Systematic Reviews (PRISMA) statement. Further, the review process was organized using the PICO (Participant, Intervention, Comparison, and Outcomes) framework, as described below:

- Participants: studies involving male patients or animals with reproductive system diseases or dysfunction.
- Intervention: studies involving patients or animals receiving RG or its extracts.
- Comparison: clinical studies involving conventional treatment, medication, placebo, and no treatment or animal models of reproductive system diseases or dysfunctions.
- Outcomes: clinical studies assessing changes in sperm quality, International Index of Erectile Function 5th version (IIEF-5), sexual satisfaction, etc. or animal experiments assessing changes in testicular weight, sperm quality, intracavernous pressure, spermatogenesis-related genes, sex hormone, inflammatory cytokines, etc.

#### Exclusion criteria

The following studies were excluded: (1) nonclinical studies or studies not involving animal experiments (review articles, case reports, letters, comments, posters, book chapters, etc.); (2) duplicates and studies with incomplete data; (3) studies not primarily focusing on RG (such as those on panax ginseng, black ginseng, American ginseng, etc.); (4) studies not primarily focusing on male reproductive diseases or dysfunction (such as those on female sexual dysfunction, female reproductive health, etc.).

### Data collection and analysis

#### Selection of studies

**Fig. 1:**
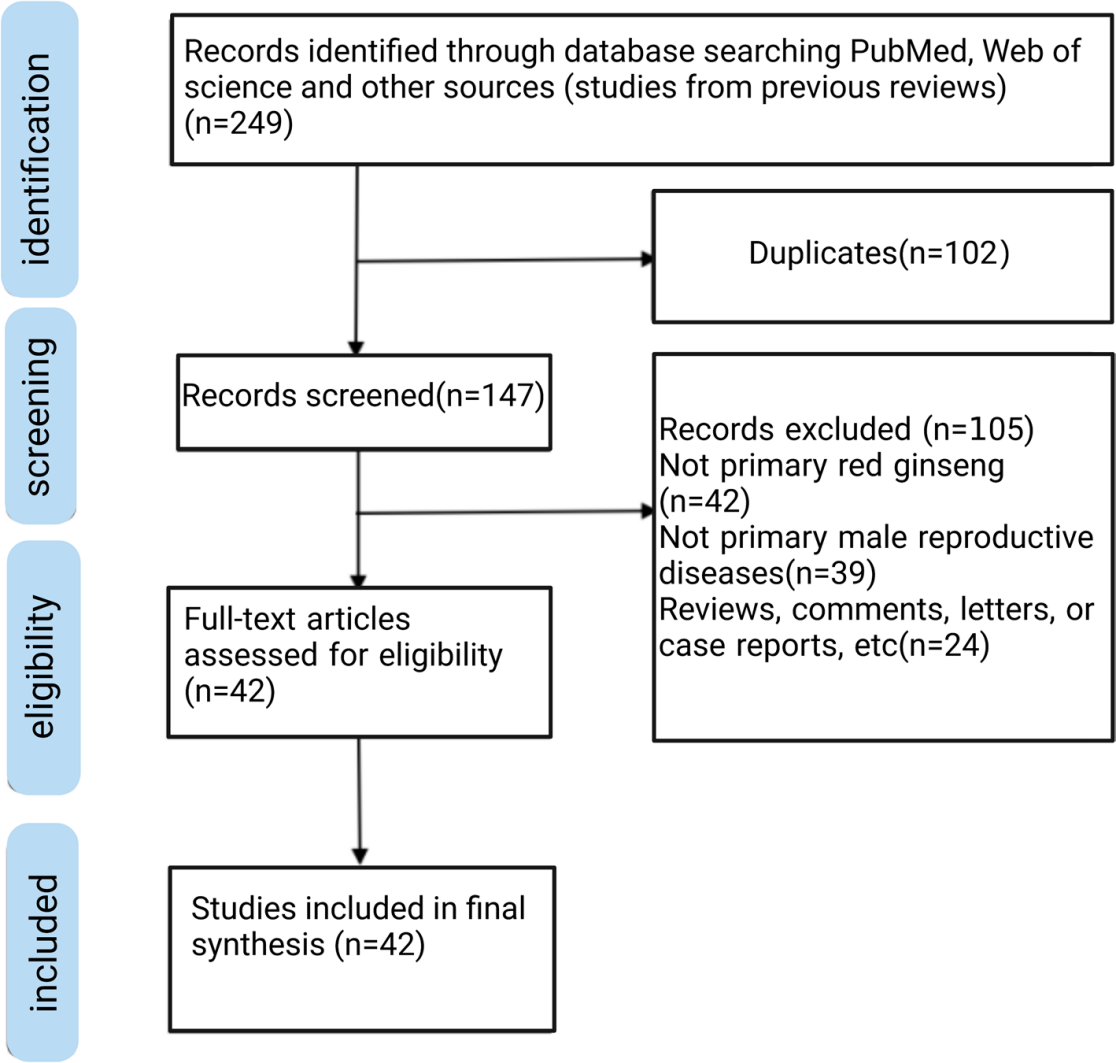
Flow chart of the study search

Two authors (Hao Wang and Bin Yan) searched for relevant articles according to the search items and then summarized the results. Original articles involving RG for MRH were included. Duplicate studies were eliminated. Some studies were excluded after analyzing the title, abstract, and the full text. The reference list of each study was also checked when necessary, to include relevant research that may have been missed in the initial search. Dissenting opinions were submitted to another author (Jiwei Zhang) for adjudication throughout the whole process (Fig. 1).

#### Data extraction

The two abovementioned authors independently extracted data in a standard format. Some of these data were the characteristics, intervention, and results. They also checked the extracted data for accuracy and completeness. Another author (Jiwei Zhang) participated in the discussions and helped resolve any disagreements.

## Results

After removing duplicates, only 147 of 249 searched studies were included in the assessment, as shown in the selection flowchart (Fig. 1). Further, after reviewing the topics, abstracts, and full text of 147 studies, 105 were excluded because they were not primary RG, primary MRH, or original articles. Finally, we included 11 clinical studies involving 289 patients who received RG and 31 animal experiments, as shown in Tables 2 and 3, respectively.

**Table 2:** Clinical trials of the effects of red ginseng on men’s reproductive health

Characteristic	Number of patients	Intervention	Results	Ref.
Age range, 25–45 years; complaint of infertility for ≥ 12 months	20	1500 mg of KRG capsules daily for 12 weeks	Sperm concentrations, motility, morphology, and viability significantly improved.	[11]
Mean age, 54 years; psychological and organic ED	22	900 mg of KRG per dose, thrice daily for 8 weeks	The mean IIEF-5 score significantly improved after 8 weeks of KRG treatment compared with that of the placebo. The mean scores of the erectile function, sexual desire, and intercourse satisfaction domains were significantly higher in the KRG group than in the placebo group after 8 weeks.	[17]
Age range, 29–61 years; mild vasculogenic ED	13	900 mg of KRG per dose, thrice daily for 3 months	In patients treated with KRG, the total and sexual satisfaction scores based on the questionnaire used in this study significantly increased, and the end-diastolic velocity in the cavernous artery decreased.	[18]
Age range, 25–70 years; mild or mild-to-moderate ED	30	600 mg of KRG per dose, thrice daily for 12 weeks	Changes in early detumescence and erectile parameters, such as penile rigidity and girth, libido, and patient satisfactions, were significantly higher in the KRG group than in other groups, statistically confirming the effect of KRG.	[19]
Mean age, 54 ± 4 years; psychological ED	28	600 or 900 mg of KRG per dose, thrice daily for 2 months	The 900 mg group had 21 patients, and the 600 mg group had 7 patients. Erectile hardness, sexual desire, and satisfaction with sex improved in 19 patients (15 from the 900 mg group and 4 from the 600 mg group).	[20]
Age range, 26–56 years; ED duration, 5.6 ± 6 years	30	600 mg of KRG per dose, thrice daily for 12 weeks	Rigidity and tumescence on erection, early detumescence, libido, and patient satisfaction were more significantly improved in the KRG group than in the placebo group.	[21]
Mean age: 43.4 ± 6.7 (Korea), 39.1 ± 10 (China), and 50.2 ± 6 (Singapore) years; organic and psychogenic ED	37	600 mg of KRG per dose, thrice daily for 3 months	The effective rates of sexual desire, erection, sexual activity, and sexual satisfaction were significantly better in the KRG group than in the placebo group.	[22]
Mean age, 53.2 ± 9.7 years; psychogenic, organic, and mixed ED	35	400 mg of KRG extract powder per dose, 2 times for 8 weeks	The primary efficacy domain (erectile function) and all secondary efficacy domains significantly improved; moreover, sexual desire, frequency, and degree of sexual desire improved.	[23]
Mean age, 45.7 ± 8.7 years; psychogenic ED	25	600 mg of KRG per dose, thrice daily for 8 weeks	The erectile hardness and maintenance were significantly better in the KRG group than in the placebo group. Post-treatment satisfaction was significantly higher in the KRG group, and it was significantly better than that in the placebo group.	[24]
Mean age, 54.3; mean baseline IIEF-5, 16.4 ± 2.9; mild ED (n = 18) and mild-to-moderate ED (n = 12)	30	1000 mg of KRG per dose, thrice daily for 12 weeks	The baseline IIEF-5 score significantly increased after KRG treatment; furthermore, erection in response to the global efficacy question; rigidity; and erection maintenance in addition to penetration were significantly better in the KRG group than in the placebo group.	[25]
Mean age, 45.1 ± 7.6; psychogenic ED	19	600 mg of KRG per dose, thrice daily for 4 weeks	Among the 19 people treated with KRG, 12 achieved better results in terms of sexual activity.	[26]

*Abbreviations*: *KRG *Korean red ginseng, *ED *Erectile dysfunction, *IIEF-5 *5-item version of the International Index of Erectile Function

**Table 3:** Animal experiments on the effect of red ginseng on men’s reproductive health

Organ	Factors/ Characteristics	Intervention medication	Animals	Result	Ref.
testes	epididymo-orchitis	KRG	Wistar rats	^a^ apoptotic of spermatogenic cells ^b^ sperm quality	[27, 28]
testes	aging	hydrogen- rich KRG	C57BL/b inbred mice	^b^ sperm quality, spermatogenesis-related genes, serum testosterone and follicle stimulating hormone	[29]
testes	aging	KRG extract	SD rats	^b^ sperm quality, antioxidant enzymes, sex hormone receptors, genes related to spermatogenesis ^b^ Cyp11a1 gene expression in testes ^a^ aberrant increase in follicle stimulating hormone and luteinizing hormone	[30–34]
testes	exposed to 2,3,7,8-Tetrachlorodiben-p-dioxin	KRG water extract	rats	^b^ survival time, sperm quality	[35, 36]
testes	exposed to busulfan	KRG extract	imprinting control region mice	^b^ testis weight, sperm quality, serum testosterone	[37]
testes	exposed to ethanol	KRG extract	C57BL/6J mice	^b^ sperm quality ^a^ aberrant decline of sex hormone	[38]
testes	exposed to zearalenone	KRG extract	SD rats	^a^ apoptotic cells in seminiferous tubules and expression of Fas and Fas-L	[39]
testes	toxicity of medicine	a formula include KRG, angelica gigas and deer antlers	specific pathogen-free SD rats	No related changes in parameters for fertility development	[40]
testes	testicular tissue injury after torsion and detorsion	KRG	SD rats	^b^ testis weight ^a^ reactive oxygen species, superoxide, and histological damage	[41]
testes	exposed to heat stress	KRG3-enriched KRG extract	SD rats	^b^ sperm quality, antioxidant enzymes, sex hormone receptors, genes related to spermatogenesis ^a^ inflammatory cytokines	[42]
testes	exposed to doxorubicin	KRG water extract	SD rats	^b^ antioxidant enzymes, sex hormone receptors, genes related to spermatogenesisa ^a^ inflammatory cytokines	[43, 44]
testes	sub-acute immobilization stress	KRG water extract	SD rats	^b^ testis and epididymis weight, sperm quality, spermatogenesis-related proteins, sex hormone receptors, and antioxidant-related enzymes	[45]
testes	none	KRG	Swiss mice	^b^ weight of testes and seminal vesicle ^b^ interstitial cells in testes	[46]
penis	none	KRG extract	rabbits, rats	^b^ relaxation of smooth muscle in the cavernous, intracavernousal pressure ^a^ contraction induced by addition of CaCl_2_	[47–49]
penis	none	9 oriental herbs (include KRG)	SD rats	^b^ the ratio of the intracavernous pressure to mean arterial pressure and the relaxation responses to acetylcholine	[50]
penis	diabetics	KRG	SD rats	^b^ intracavernous pressure, glutathione ^a^ malondialdehyde levels	[51]
penis	metabolic syndrome	KRG	Wistar rats	^b^ ratio of intracavernous pressure to mean arterial pressure ^b^ cyclic guanosine monophosphate in corpus cavernosum	[52]
penis	none	ginsenoside KRG1 of KRG	mice	^b^ incidence of copulatory behavior	[53]
prostate	benign prostatic hyperplasia	KRG oil	SD rats	^a^ prostate overweight, dihydrotestosterone and 5α-reductase	[54]
prostate	bacterial prostatitis and treated with ciprofloxacin	KRG	Wistar rats	^a^ apoptotic cells in prostate	[55]
prostate	prostate hyperplasia	KRG water extract	SD rats	^a^ activation of androgen receptor, prostate overgrowth and epithelial thickening	[56]
prostate	chronic prostatitis	KRG	Wistar rats	^a^ inflammatory cytokines	[13]

*Abbreviations*: *KRG *Korean red ginseng, *SD *Sprague dawley

^a^represents the inhibition effect, ^b^represents the promotion effect

### Clinical studies on the effect of red ginseng on men’s reproductive health

Among 11 clinical studies included (Table 2), 10 were randomized controlled trials of RG in erectile dysfunction (ED) [11, 17–26] and 1 was a randomized controlled trial of RG in male infertility (MI) [11]. Varying doses of RG were used for treating male reproductive disorders. The daily dose of Korean RG ranged from 800 mg to 3000 mg, and the duration of treatment ranged from 4 weeks to 3 months. Five of these studies used the dose of 600 mg thrice daily, whereas two used the dose of 900 mg thrice daily. The results of the included studies revealed that 1500 mg of RG orally administered daily for 12 weeks significantly improved sperm concentration, viability, and morphology in the patients. Another 10 studies consistently reported that RG enhanced erectile function and alleviated male sexual dysfunction in patients. Although these clinical studies lacked consistency in the efficacy indicators and preferred subjective patient perceptions, they revealed that RG improved the IIEF-5 scores and sexual life satisfaction. Notably, in the study by Kim et al. [20], 21 patients received 900 mg of RG thrice daily, whereas 7 received 600 mg RG thrice daily. In their study, factors such as erectile hardness, sexual desire, and satisfaction with sex improved in 19 (67%) patients, of which 15 were from the 900 mg group and 4 from the 600 mg group. Given the large difference in the rates between the two groups, Kim et al. could not confirm which group was more effective. Further clinical studies are required to validate the optimal use of RG in male reproductive diseases. Nevertheless, the abovementioned 11 studies reported no significant serious adverse effects.

### Animal experiments to verify the mechanisms of red ginseng on men’s reproductive health

The 31 studies in Table 3 showed that the effects of RG on MRH are mainly achieved by acting on target organs such as the testis, penis, and prostate [13, 27–56]. RG is beneficial to MRH through various mechanisms of action. The majority of these RG studies (20 articles) focused on spermatogenic dysfunction, particularly testicular function injury caused by RG improvement exposure factors. The choice of medicine was based more on RG extract. However, most studies did not specify the specific extract ingredients. RG is generally used as a health food because of the richness in ginsenosides, volatile oils, polysaccharides, amino acids, peptides, and other components with high nutritional values. Hence, our review results suggest the need for further exploration on the specific component of RG that is beneficial to male spermatogenic function. Nonetheless, our results also suggest that Korean RG has a restorative effect on ED caused by different diseases [51, 52]. Moreover, given that prostate was one of the target organs of RG in these studies, RG and its extracts were also being related to the regulation of prostate function.

## Discussion

With the increasing prevalence of MI, ED, and prostate diseases in recent years [57–59], these diseases pose serious threats to patients’ quality of life, mental health, and family relations. Accordingly, more patients are actively seeking relevant treatment. Based on our clinical research evidence, RG—a safe functional food—can effectively improve erectile function in patients with ED, restore semen quality in patients with MI, and improve MRH. Further, based on the relevant evidence from animal experiments, RG’s potential mechanism of action on testis, penis, and prostate gland was clarified. To the best of our knowledge, no study in the relevant literature has analyzed various studies on RG utilization for MRH to date [60, 61]. The potential mechanisms of RG in the treatment of MI, ED, and prostate disease need to be summarized and analyzed to clarify the value of RG in MRH and to provide a reference for future clinical applications.

### Red ginseng and male infertility

MI refers to the inability of the female partner to conceive naturally because of male factors after the couple has had regular sex for > 1 year without using any contraception [62, 63]. The causes of MI are complex, and they include aging [64], occupational exposure [65], drug injury [66], infection, and other self-inflicted diseases [67]. RG monotherapy somewhat reversed reproductive damage in animal models exposed to tetrachlorodibenzo-p-dioxin, zearalenone, and RG monotherapy in combination with numerous drugs to mitigate pharmacogenically induced reproductive toxicity [35, 36, 39]. For example, ciprofloxacin can lead to a decrease in testicular weight, sperm quality, and testosterone level in patients with testicular inflammation—an important factor in sperm arrest and testicular atrophy [68]. However, ciprofloxacin combined with RG has been reported to improve sperm quality and reduce apoptosis indicators in Wistar rats [28]. Animal experiments have reported that the combination of RG with antitumor drugs, busulfan and doxorubicin, also attenuates drug toxicity–induced damage to the reproductive system [37, 43, 44]. Although the exact mechanism is unclear, an increasing number of studies have tended to attribute RG’s mechanism of alleviation of pharmacogenic reproductive toxicity to antioxidant effects [43, 44].

**Fig. 2:**
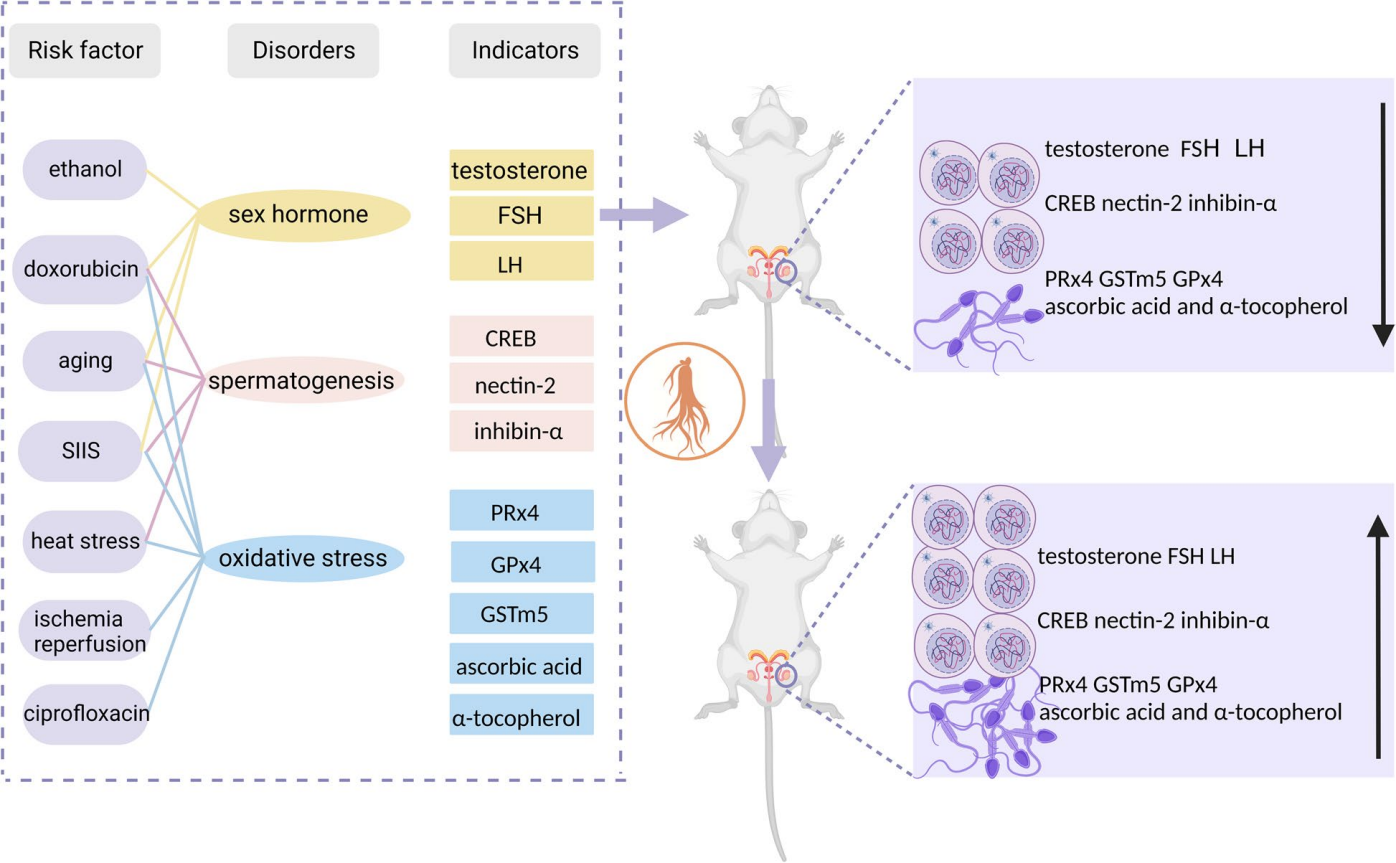
Mechanism of the therapeutic effect of red ginseng on male infertility. Abbreviations: FSH, follicle stimulating hormone; LH, luteinizing hormone; CREB, CAMP responsive element binding protein; PRx4, peroxiredoxin 4; GPx4, glutathione peroxidase 4; GSTm5, glutathione S-transferase mu 5, SIIS, subchronic intermittent immobilization stress. (Created with BioRender.com)

Multifunctional oxidoreductases, such as glutathione peroxidase 4 (GPx4), glutathione S-transferase mu 5 (GSTm5), and peroxiredoxin 4 (PRx4), are involved in the spermatogenic pathway; when the expression of these proteins in testicular cells is downregulated, oxidative damage and cell death may occur [69–71]. RG—a natural antioxidant—increased the expression of PRx4, GSTm5, and GPx4 in animal models while decreasing the serum levels of reactive oxygen species (ROS), as confirmed by numerous relevant studies [30, 31, 41] (Fig. 2). In addition, RG normalized the recovery of antioxidants such as glutathione-S-transferase (GST), ascorbic acid, and α-tocopherol in the testes of aging rats and prevented oxidative damage to the testicular tissue by reducing the production of nicotinamide adenine dinucleotide phosphate oxidase and superoxide [29–34].

Key biomolecules such as inhibin-α, CAMP responsive element binding protein 1 (CREB-1), and nectin-2 are involved in testicular function and are considered to be sex hormone pathway molecules associated with spermatogenesis [72]. Inhibin-α, a gonadal glycoprotein, is essential in regulating the secretion of follicle stimulating hormone (FSH) [73]. CREB-1 is expressed during the mitotic phase of spermatogenesis and the differentiation phase of spermatogenesis, thereby playing an important role in spermatogenesis [74]. Furthermore, nectin-2 promotes support-support cells or support-mature germ cells in the seminiferous tubules of the testis in close contact and contributes to the development of mature spermatozoa in the seminal vesicle epithelium [74]. The impaired expression of these genes induced by doxorubicin, aging, subchronic intermittent immobilization stress, and heat stress can be reversed by RG, restoring spermatogenic function in rat testes (Fig. 2) [29–34, 42–45].

Moreover, RG may improve sperm quality by regulating the production of testosterone, FSH, and luteinizing hormone (LH) [29–34, 38, 43–45]. Testosterone is essential for the function and maintenance of the structure of the male secondary gonads and is related to spermatogenesis [75, 76]. Meanwhile, LH promotes testosterone synthesis by testicular mesenchymal cells, and FSH acts on the FSH receptor in supporting cells to aid spermatogenesis [77]. In animal experiments, RG and its extracts significantly attenuated spermatogenesis disorders caused by sex hormone alterations resulting from various factors (e.g., drug damage, aging, ethanol, and subchronic intermittent immobilization stress), elevated testosterone levels, and mitigated the aberrant increase in FSH and LH levels (Fig. 2) [29–34, 38, 43–45].

### Red ginseng and erectile dysfunction

**Fig. 3:**
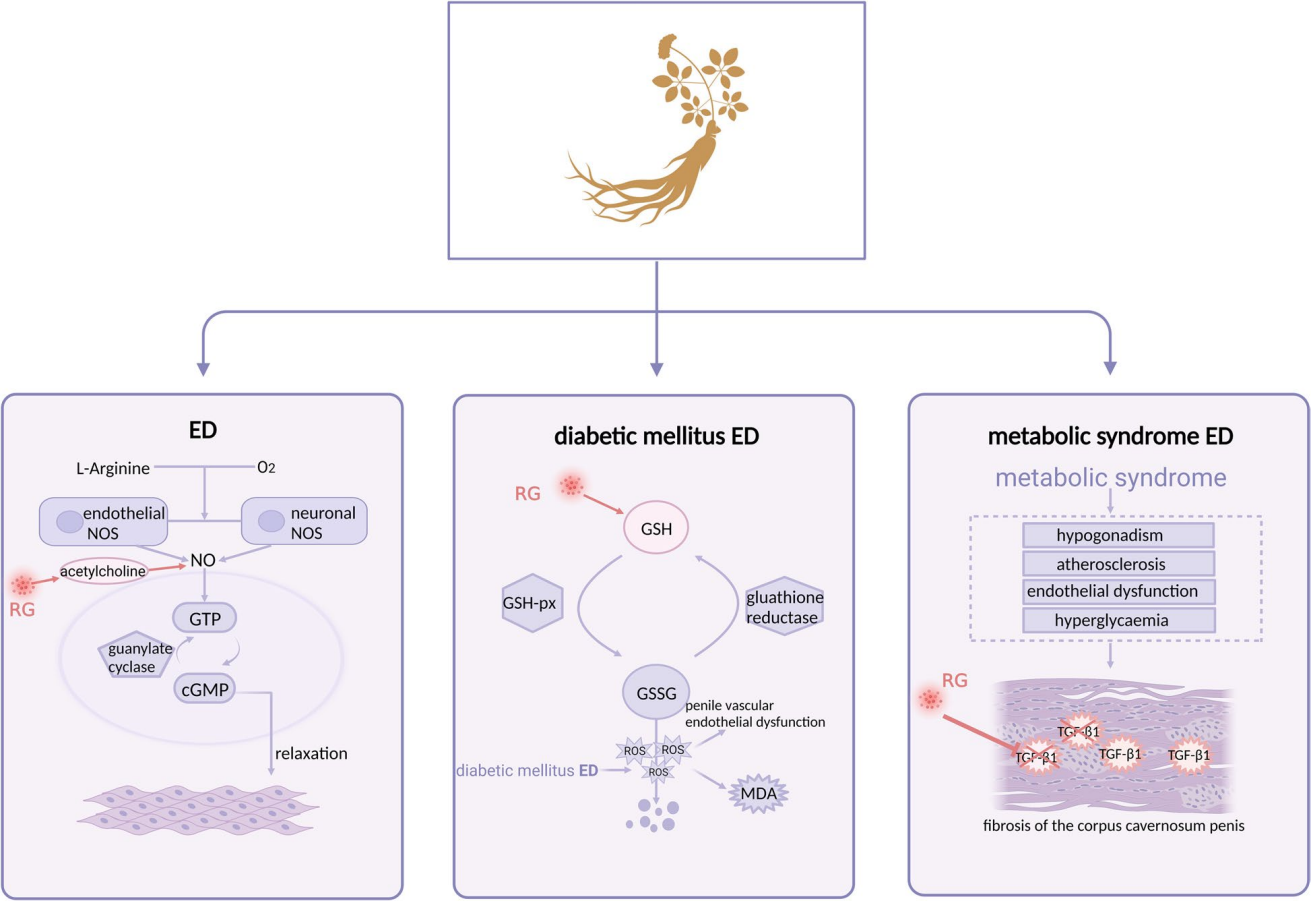
Mechanism of the therapeutic effect of red ginseng on erectile dysfunction. Abbreviations: NOS, nitric oxide synthase; NO, nitric oxide; GTP, guanosine triphosphate; cGMP, cyclic guanosine monophosphate; GSH, glutathione; GSH-px, glutathione peroxidase; GSSG, glutathione oxidized; MDA, malondialdehyde; ROS, reactive oxygen species; TGF-β1, transforming growth factor-β 1. (Created with BioRender.com)

ED is a common sexual dysfunction, in which men cannot consistently obtain and maintain an erection sufficient to complete a satisfactory sexual intercourse [78, 79]. Its common risk factors include cardiovascular disease, including aging, diabetes, and metabolic syndrome [80–82]. Activation of the smooth muscle of the penile corpus cavernosum requires the action of endothelial cell relaxing factor or nitric oxide (NO) [83], which is an essential physiological signal for penile erection; diseases that reduce NO synthesis or release in the erectile tissue are usually associated with ED [84]. Nitric oxide synthase (NOS), which includes neuronal NOS and endothelial NOS, uses L-arginine as a substrate to generate NO from oxygen; acetylcholine may also facilitate NO production and release. The released NO activates guanylate cyclase in the smooth muscle cytoplasm to produce cyclic guanosine monophosphate (cGMP) [85]. RG may act by releasing or enhancing the endothelial cell relaxing factor and increasing acetylcholine-induced rabbit cavernous muscle relaxation, which may be mediated by NO and/or cGMP [49] (Fig. 3).

In the treatment of diabetes mellitus–associated ED, Ryu et al. used malondialdehyde (MDA) as an indicator of oxidative stress and measured the glutathione (GSH) levels to confirm the antioxidant effect of RG on the cavernous body of rats with diabetic mellitus–associated ED [51]. These rats had significantly higher MDA levels and significantly lower GSH levels than normal rats, but these outcomes were reversed after RG treatment [51]. Excessive ROS causes lipid peroxidation of unsaturated fatty acids on mitochondrial membranes to form lipid peroxides and then produce MDA, resulting in increased MDA content in mitochondria [86]. MDA can quantify free radical damage to cells. Glutathione peroxidase catalyzes the change in GSH, forming oxidized glutathione; it also promotes peroxide decomposition and protects the structure and function of cell membranes from interference and damage caused by peroxides (Fig. 3) [87]. Under long-term high glucose induction in diabetes, ROS is excessively produced in the organism and mitochondria, and the activity of free radical scavenging enzymes such as GSH peroxidase decreases as a result of non-enzymatic glycosylation; the scavenging of free radicals such as oxygen and hydrogen peroxide is also reduced, leading to further accumulation of ROS in the body and subsequently aggravating oxidative stress damage and organ dysfunction [87]. This mechanism supports an imbalance between free radical production and scavenging in the spongiosa of diabetic rats, and RG plays a role in the antioxidant activity [51].

Metabolic syndrome may cause vascular endothelial dysfunction through multiple pathways. With regard to improving erectile function in ED rats with metabolic syndrome, penile cavernous fibrosis progresses in vascular-derived ED. In addition, the expression of transforming growth factor-β 1 (TGF-β1), a molecular marker of fibrosis in fractionated vascular ED, induces damaged-tissue repair by aggregating fibroblasts in areas of ischemic-tissue damage and promoting the production of collagenous connective tissue [88]. In the study by Kim et al. [52], the proportion of penile smooth muscle cells in rats suffering from metabolic syndrome was significantly reduced, whereas that with RG treatment was close to that of normal controls and inhibited TGF-β1 expression in the penile corpus cavernosum (Fig. 3).

### Red ginseng and prostate diseases

**Fig. 4:**
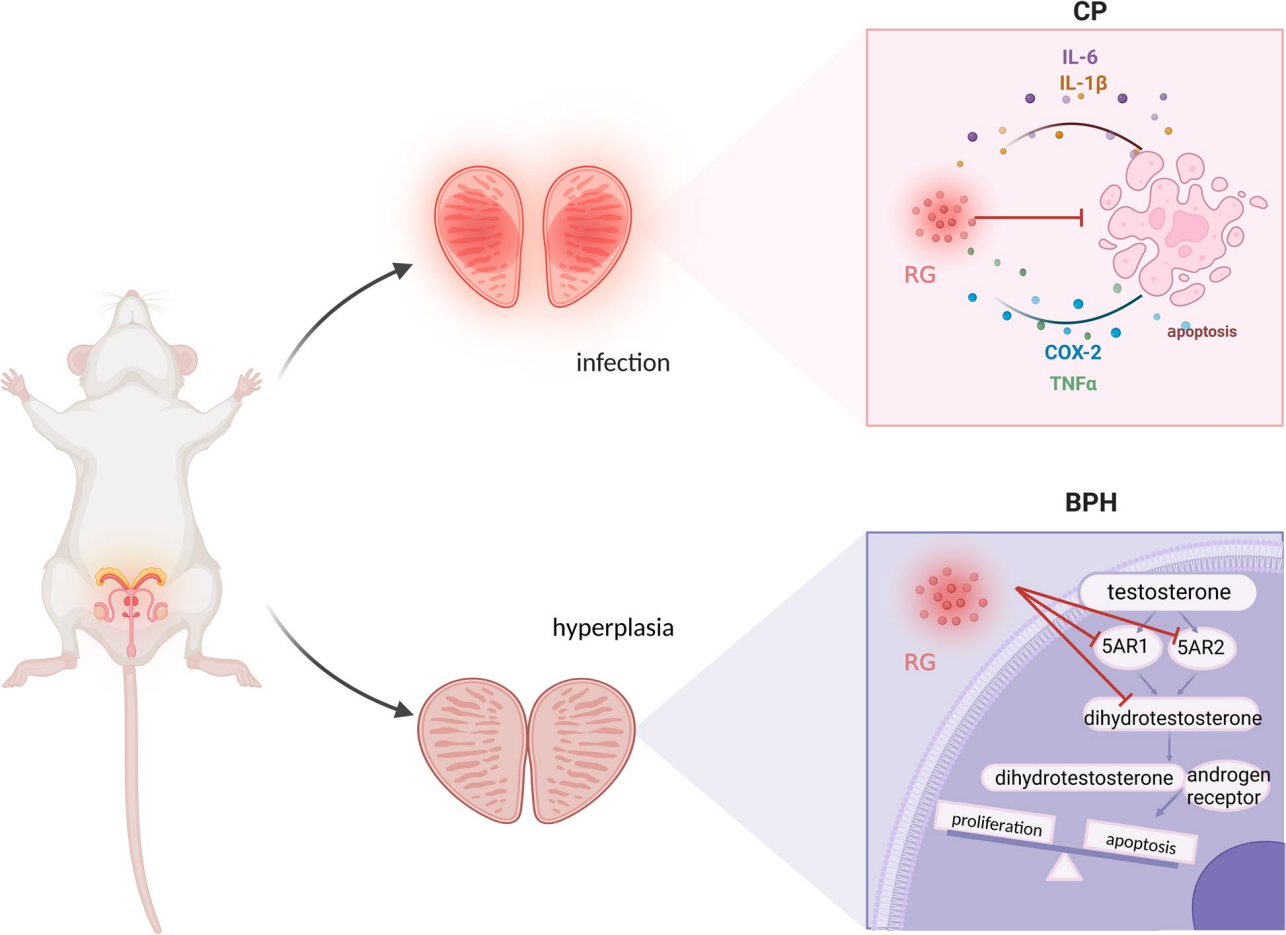
Mechanism of the therapeutic effect of red ginseng on prostate diseases. Abbreviations: IL-6, interleukin-6; IL-1β, interleukin-1β; COX-2, cyclooxygenase-2; TNF-α, tumor necrosis factor-α; 5AR, 5α-reductase. (Created with BioRender.com)

Prostate disease is a common disease in adult men, usually related to prostatitis, benign prostatic hyperplasia (BPH), and prostate cancer. The results of our study revealed that chronic prostatitis (CP) and BPH are closely related to RG. CP represents a group of syndromes caused by multiple factors, mainly pain or discomfort in the pelvic region and lower urinary tract symptoms; further, it is subdivided into bacterial and nonbacterial types [89]. Inflammatory diseases of the reproductive system, such as epididymitis and other accessory gonad infections caused by *Escherichia coli*, may cause prostate congestion and edema, inducing bacterial prostatitis [90]. RG can reduce prostate weight gain in rats with acute epididymitis and improve the low semen quality caused by ciprofloxacin administration for bacterial prostatitis [27, 28]. Various cytokines, including tumor necrosis factor-α, vascular endothelial growth factor, interleukin-6, interleukin-1β, and cyclooxygenase-2, are involved in the inflammatory response [91]. RG inhibited the expression of these inflammatory factors in rats and subsequently reversed the apoptosis caused by CP (Fig. 4) [13, 55].

BPH is a disease characterized by histological hyperplasia of the interstitial and glandular components of the prostate, affecting mostly middle-aged and older adult men; consequently, the prostate enlarges, causing urodynamic bladder outlet obstruction and clinical symptoms mainly in the lower urinary tract [92]. Testosterone increases the risk of BPH and worsens the lower urinary tract symptoms [93]. Through 5α-reductase (5AR) in the prostate, testosterone is converted into activated dihydrotestosterone, which then binds to the androgen receptor in prostate cells, inducing transcriptional activation of target genes and apoptosis and proliferative imbalance in prostate cells, leading to BPH [94]. Therefore, the inhibition of androgen production and blockade of androgen receptor signaling may be important strategies for BPH treatment [95]. In rats, daily injection of Korean RG water extract prevented testosterone-induced prostate overgrowth and epithelial cell thickening and inhibited androgen receptor activation [56]. In vivo studies have also shown an 18% reduction in prostate weight and downregulation of the expression of dihydrotestosterone, 5AR2, and 5AR1 in BPH rats receiving RG oil [54] (Fig. 4). In addition, increased apoptosis helps ameliorate the hyperproliferation of prostate cells. Both B-cell lymphoma 2 (Bcl-2, an anti-apoptotic protein) and Bcl-2-associated X protein (Bax, a pro-apoptotic protein that induces apoptosis) [96] aid in regulating the mitochondrion-mediated apoptotic pathway. Furthermore, changes in BPH were associated with increased Bcl-2 levels and decreased Bax levels in the same tissue [97], but these effects were reversed after RG extract treatment; hence, RG may inhibit prostate tissue overexpression by inducing apoptosis [54].

## Conclusion

To the best of our knowledge, this study is the first to review the effects of RG on MRH, providing a new and unique perspective on the treatment of related diseases. For MI, the research on RG tends to be animal experiments, mainly concentrating on oxidative stress, sex hormones, and related indicators of spermatogenesis. Further studies are required to elucidate the upstream mechanisms and verify the mechanism of action of RG in regulating the related specific pathways. Although several clinical studies have analyzed the use of RG for ED, the data on the included participants and efficacy evaluation indexes significantly differ among the clinical trials. Thus, potential mechanisms to improve erectile function should be explored further. Moreover, given that the use of RG in prostate diseases remains unknown, the efficacy and specific mechanism involved in RG treatment for such diseases should be examined in the future.

### Supplementary Information

**Additional file 1.**

**Additional file 2.**

**Additional file 3.**

**Additional file 4.**

## Data Availability

All data generated or analyzed during this study are included in this published article.

## Abbreviations

RG: Red ginseng
MRH: Men’s reproductive health
ED: Erectile dysfunction
MI: Male infertility
GPx4: Glutathione peroxidase 4
GSTm5: Glutathione S-transferase mu 5
PRx4: Peroxiredoxin 4
ROS: Reactive oxygen species
GST: Glutathione-S-transferase
CREB-1: CAMP responsive element binding protein 1
FSH: Follicle stimulating hormone
LH: Luteinizing hormone
NO: Nitric oxide
NOS: Nitric oxide synthase
MDA: Malondialdehyde
GSH: Glutathione
TGF-β 1: Transforming growth factor-β 1
BPH: Benign prostatic hyperplasia
CP: Chronic prostatitis
5AR: 5α-reductase
Bcl-2: B-cell lymphoma 2
Bax: Bcl-2-associated X protein
