# Supplementary material for: The role of red ginseng in men’s reproductive health: a literature review
Source: Basic Clin Androl. 2023 Oct 26;33:27. doi: 10.1186/s12610-023-00203-0 (PMC10601307; doi:10.1186/s12610-023-00203-0)
Supplement: Supplementary file 1 — Additional file 1. [file 12610_2023_203_MOESM1_ESM.pdf]

;enago
Author First, Quality First
www.enago.cn

CERTIFICATE OF EDITING

This is to certify that the paper titled Role of red ginseng in men's reproductive health: A literature review commissioned to us by Hao Wang has been edited for English language, grammar, punctuation, and spelling by Enago, the editing brand of Crimson Interactive Consulting Co. Ltd. under 高级论文润色服务.

ISO 17100:2015
Translation Service Providers

ISO 27001:2013
Information Security Management System

ISO 9001:2015
Quality Management System

Issued by:

Enago, Crimson Interactive (Beijing) Consulting Co., Ltd.
Room 3217, Cyber Tower A,
No. 2, Zhongguancun South Street,
Haidian District, Beijing 100080

Disclaimer : The intent of the author's message has been preserved during the editing process. The author is free to accept or reject our changes in the document after reviewing our edits. This certificate has been awarded at the time of sharing the final edited version (full file or sections of the file) with the author. Enago does not bear any responsibility for any alterations done by the author to the edited document post 7 7月 2023.

Japan	www.enago.jp, www.ulatus.jp, www.voxtab.jp	Russia	www.enago.ru
Taiwan	www.enago.tw, www.ulatus.tw	Arabic	www.enago.ae
China	www.enago.cn, www.ulatus.cn	Turkey	www.enago.com.tr
Brazil	www.enago.com.br, www.ulatus.com.br	S. Korea	www.enago.co.kr
Germany	www.enago.de	Global	www.enago.com, www.ulatus.com, www.voxtab.com

About Crimson:
Crimson Interactive Consulting Co. Ltd. is one of the world's leading academic research support services. Since 2005, we've supported over 2 million researchers in 125 countries with their publication goals.
